# Performance of Fly Ash-Modified Self-Compacting Concrete Under Realistic Field Curing Conditions: A Combined Maturity and Microstructural Analysis

**DOI:** 10.3390/ma19101996

**Published:** 2026-05-12

**Authors:** Sefa Talay, Ahmet Ferhat Bingöl, Dilek Okuyucu, Burak Gedik, Muhammet Şahin

**Affiliations:** 1 Technical Sciences Vocational School, Muş Alparslan University, 49250 Muş, Türkiye; 2 Department of Civil Engineering, Atatürk University, 25240 Erzurum, Türkiye; afbingol@atauni.edu.tr; 3 Department of Civil Engineering, Erzurum Technical University, 25100 Erzurum, Türkiye; okuyucu@erzurum.edu.tr (D.O.); burak.gedik@erzurum.edu.tr (B.G.); muhammet.sahin65@erzurum.edu.tr (M.Ş.)

**Keywords:** self-compacting concrete, fly ash, maturity method, cold weather, hydration, microstructure, SEM, XRD, strength development

## Abstract

**Highlights:**

- Early-age temperature critically governs strength development of SCC under variable curing conditions.
- Maturity method (FHP model) provides reliable strength prediction under realistic field conditions.
- Fly ash incorporation alters hydration kinetics and improves long-term microstructural development.
- Field curing conditions must be considered to accurately evaluate concrete performance.
- Heating significantly enhances strength development under cold-weather conditions.

**Abstract:**

This study examines how fly ash-modified self-compacting concrete (SCC) behaves during curing under real conditions, focusing on changes in temperature and heat during the first days. Unlike typical lab tests at steady temperatures, three settings were used to copy real-life conditions: summer, winter with heating, and winter without heating. Temperature changes were tracked with built-in temperature sensors. Concrete maturity was calculated using a standard method (the Freiesleben-Hansen and Pedersen approach in ASTM C1074). The results show that heat in the first 72 h affects the maturity and strength of the concrete. After 7 days, strengths were measured as 32.7 MPa in summer, 27.2 MPa in winter-heated, and 15.7 MPa in winter-unheated settings. Predictions of strength based on maturity closely matched the measured values, proving that this approach works well in real settings. Examining the concrete’s structure with SEM and XRD tools showed that fly ash alters how the concrete forms and becomes denser, while lower temperatures slow key reactions, making the material more porous. These results show why early heat control matters. The maturity approach helps reliably estimate in situ strength and guide mix design for real projects.

## 1. Introduction

Self-compacting concrete (SCC) is widely adopted in modern construction because of its high flowability, passing ability, and elimination of vibration requirements. The addition of mineral admixtures, such as fly ash, significantly influences SCC’s rheology and long-term strength by affecting hydration-driven microstructural changes [1,2].

Concrete production in cold weather poses major engineering challenges. Hydration kinetics depend on temperature. Low temperatures slow cement hydration, delay early strength, and may threaten construction safety, especially during formwork removal and initial loading [3,4,5]. Regions with variable weather and large day–night temperature changes add further complications. Concrete here is exposed to non-isothermal curing, making strength estimation and curing management harder [6].

The maturity method estimates in situ concrete strength by linking temperature–time history with strength gain. Introduced by Saul [7] and refined by Hansen and Pedersen, it is now standardized in ASTM C1074 [8]. Of available models, the Arrhenius-based Freiesleben-Hansen and Pedersen (FHP) model performs well across wide temperature ranges [9,10,11]. However, most research relies on constant laboratory conditions. Real-world maturity-based strength prediction is still underexplored [10,12,13].

Temperature strongly affects hydration and microstructural change in cementitious systems. In fly ash-modified concrete, pozzolanic reactions are more sensitive to cold, leading to delayed hydration, slower strength gain, and changes in the final microstructure [14,15,16,17,18]. Although fly ash is common in SCC, maturity-based strength prediction for such mixes under real, changing field temperatures remains to be studied.

SEM and XRD are key for studying hydration products and phase changes [19,20], yet only a few studies combine maturity-based strength prediction with microstructural evaluation under true atmospheric curing.

Despite the growing body of research on self-compacting concrete (SCC), several important gaps remain regarding the performance of fly ash-modified SCC under realistic field curing conditions. In particular, limited studies have investigated strength development under actual atmospheric temperature–time histories, especially in cold-weather environments. In addition, the applicability of maturity-based strength prediction methods under fluctuating, non-isothermal field conditions has not been adequately validated. Furthermore, integrated studies combining mechanical performance with microstructural characterization, such as scanning electron microscopy (SEM) and X-ray diffraction (XRD), remain scarce. Addressing these gaps is essential for improving the reliability and practical application of SCC in cold-weather construction.

This study presents a field-scale experiment on SCC under realistic seasonal curing. Concrete specimens were cast in summer, winter-heated, and winter-unheated conditions to capture real temperature variations. Maturity indices were calculated from temperature records, and maturity method predictions were tested. SEM and XRD analyses tracked hydration and microstructure, which were then compared with compressive strength results.

This study aims to provide a reliable method for predicting strength under non-isothermal field conditions. It also seeks to link temperature history, hydration, and mechanical performance in SCC with fly ash.

## 2. Materials and Methods

### 2.1. Materials

In this study, CEM II/A-LL 42.5 R Portland composite cement, compliant with EN 197-1, was used as the binder (see Table 1 for properties) [21]. To improve workability and long-term performance, Class F fly ash from Emba Elektrik Üretim A.Ş. (Adana-Yumurtalık, Türkiye) was used at a replacement level of 25% of the total binder content, in line with SCC mixture guidelines [14]. Fly ash properties are listed in Table 2.

**Figure 1:**
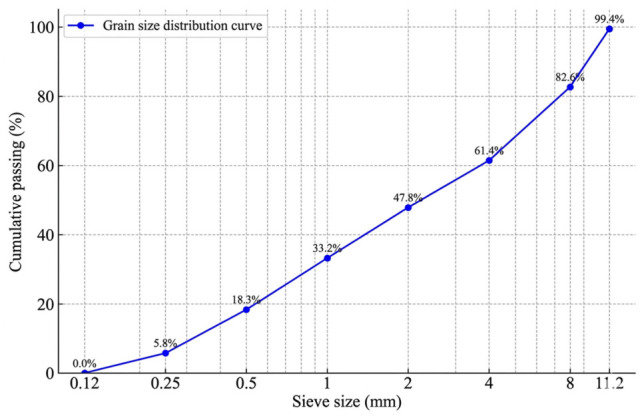
Aggregate grading of SCC mixtures.

River sand (0–4.75 mm) was used as the fine aggregate, while river aggregate (4.75–12 mm) was used as the coarse aggregate. The fine and coarse aggregates constituted 55% and 45% of the total aggregate content by mass, respectively. The specific gravities of the fine and coarse aggregates were 2.34 and 2.45 g/cm^3^, respectively, with corresponding water absorption values of 3.47% and 2.88%. The aggregate grading is shown in Figure 1.

A polycarboxylate-based superplasticizer (CHRYSO Fluid Optima H 439) was used at a dosage of 1% of the binder content to ensure the required flowability of SCC [22]. Mixing water conforming to TS EN 1008 was used throughout the study [23].

### 2.2. Mix Design

The SCC mixture was optimized through preliminary trial studies based on proportions recommended in the literature, and the final mix achieving the C40 strength class was selected from eight trial mixtures. The water-to-binder ratio was fixed at 0.45, with a total binder content of 500 kg/m^3^ (375 kg CEM II/A-LL 42.5 R cement and 125 kg Class F fly ash). The mixture proportions are presented in Table 3.

Fresh concrete properties were evaluated using slump flow [24], V-funnel [25], and L-box [26] tests in accordance with EFNARC (2005) guidelines [27]. The specified limits and corresponding test results are presented in Table 4. The results satisfied the EFNARC requirements and confirmed the self-compacting performance of the mixture.

### 2.3. Experimental Environments and Specimen Preparation

**Figure 2:**
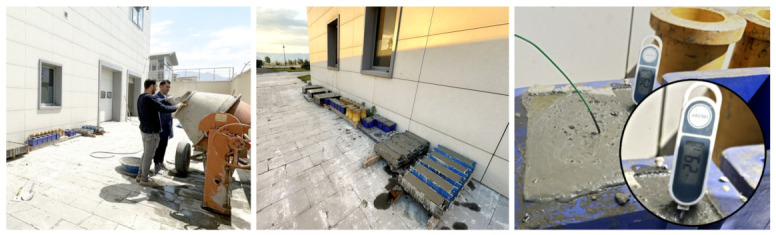
The environment where the summer group specimens were prepared and stored.

**Figure 3:**
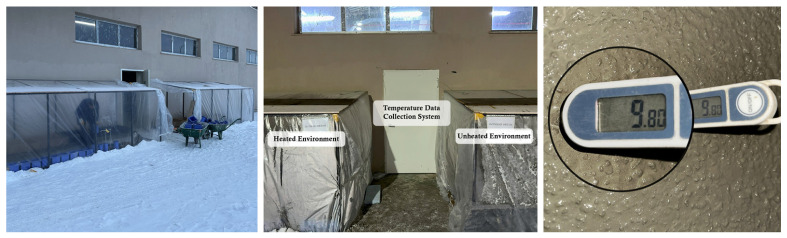
The environments where the winter group specimens were prepared and stored.

This study aims to evaluate how varying curing conditions, representative of summer and winter environments, affect the properties of reinforced concrete slabs. To achieve this, slabs were produced and cured under different regimes: atmospheric water-curing for summer specimens (Figure 2), and heated and unheated greenhouse plastic enclosures for winter specimens (Figure 3).

In the winter-heated condition, the specimens were placed inside a greenhouse-type enclosure with an internal volume of approximately 21.5 m^3^. Heating was provided by an oil-filled radiator (Beko BRI 11D, İstanbul, Türkiye) with a nominal power of 2300 W. The system was operated under thermostatic control, maintaining the internal air temperature at 10 ± 2 °C during the first 72 h of curing. The enclosure temperature was continuously monitored using embedded thermocouples to ensure stable thermal conditions. After this period, the specimens were removed from the heated enclosure and exposed to ambient atmospheric conditions.

To build on this approach, the experimental setup was designed to simulate realistic field conditions, in which concrete is exposed to fluctuating atmospheric temperatures rather than in constant laboratory environments. Such variations are known to significantly influence hydration kinetics and strength development, particularly in continental climates.

Continuing the experimental methodology, for temperature monitoring, reinforced slab specimens (700 × 700 × 150 mm) and cube specimens (150 × 150 × 150 mm) were prepared for each curing condition. K-type thermocouples were embedded to record internal concrete temperature, reinforcement surface temperature, formwork temperature, and ambient temperature over a period of 7 days. This configuration also enabled evaluations of the influence of specimen geometry and volume-to-surface ratio on thermal behavior.

Following temperature monitoring, compressive strength tests were conducted on three cube specimens for each curing condition at 7 and 28 days, with average values reported. The tests were performed in accordance with TS EN 12390-3 and ASTM C39 standards [28,29], using a UTEST 3000 kN Compression Testing Machine (Ankara, Türkiye).

Microstructural analyses were carried out using a ZEISS Sigma 300 Scanning Electron Microscope (Jena, Germany). Secondary electron (SE) imaging was employed to examine the morphology and hydration products of the samples. SEM imaging was performed for all curing conditions at an accelerating voltage of 5 kV, with magnifications of approximately 1.0 k×, 5.0 k×, and 10.0 k×, enabling a comparative evaluation of the microstructural characteristics.

In addition to qualitative observations, SEM images were quantitatively analyzed using ImageJ software to determine the pore area fraction. SEM micrographs acquired at 5.0 k× magnification were converted to 8-bit grayscale and processed using a constant threshold range (0–70) to segment pore regions. This threshold was selected to isolate the darkest regions corresponding to pores while minimizing the inclusion of hydration products. The same threshold and magnification were consistently applied to all samples to ensure comparability. The pore area fraction was calculated as the ratio of black pixels to the total image area. It should be noted that this method provides a two-dimensional, image-based pore area fraction rather than absolute volumetric porosity.

Phase identification was conducted using a PANalytical Empyrean X-ray Diffractometer to determine (Almelo, Netherlands) the crystalline phases and structural changes in the specimens.

The experimental results were correlated with maturity indices calculated using the Arrhenius-based Freiesleben-Hansen and Pedersen approach [10], and the predictive performance of the Hansen–Pedersen model was evaluated.

### 2.4. Hydration Process and Temperature Monitoring

**Figure 4:**
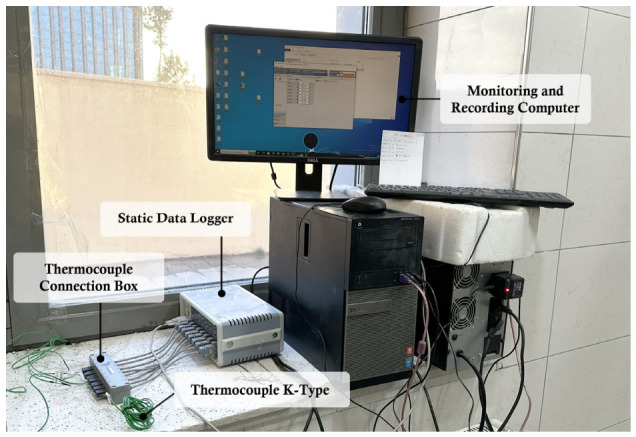
The environment and devices used for temperature monitoring.

Temperature measurements for slab specimens produced under both summer and winter conditions were carried out using K-type thermocouples placed within the slab concrete, inside cube specimens, and in the ambient environment. During winter conditions, additional thermocouples were installed inside both heated and unheated enclosure tents to monitor internal air temperature. Measurements were recorded continuously for at least 72 h; subsequently, the specimens monitored under winter conditions were exposed to ambient conditions, and temperature monitoring was continued for 28 days. All thermocouples were connected to a static data acquisition system via a junction box, as shown in Figure 4.

Following data collection, the temperature–time history was used to calculate the maturity index in accordance with ASTM C1074, enabling the evaluation of early-age strength under non-isothermal curing conditions.

### 2.5. Maturity and Strength Prediction

Strength prediction using the maturity method consists of two main steps. First, the temperature–time history of concrete is converted into a maturity index or equivalent age (EA) using Equation (1). Second, the maturity values are correlated with compressive strength, allowing the calibration of a predictive function for strength under field conditions. Various models have been proposed to describe the maturity–strength relationship [10,11], with Arrhenius-based approaches providing more reliable results under variable-temperature conditions. In this study, maturity was calculated using the Freiesleben-Hansen–Pedersen (FHP) model in accordance with ASTM C1074. Temperature data obtained from embedded thermocouples placed at the internal concrete core, reinforcement surface, formwork surface, and ambient environment were recorded at 10 min intervals. The maturity index for each time step was calculated using Equation (1).

(1) $$M(T_{\Gamma })=\sum \exp\left[-\frac{E}{R}\left(\frac{1}{273+T}\right)-\left(\frac{1}{273+T_{r}}\right)\right].\Delta t$$

Here, *M* represents the maturity index at the reference temperature (hours), *T* is the measured temperature (°C), *T_r_* is the reference temperature (20 °C), *E_a_* is the activation energy (42,000 J/mol), *R* is the universal gas constant (8.314 J/mol·K), and ∆*t* is the time interval. This formulation accounts for the temperature dependency of hydration kinetics and enables accurate maturity evaluation under non-isothermal conditions.

Temperature monitoring was conducted continuously for 28 days, and maturity values were calculated directly from the measured temperature–time history, without applying any isothermal extension beyond the experimental period.

The relationship between maturity and compressive strength was defined using the Hansen–Pedersen model (Equation (2)) [8,10]. Model parameters were determined by least-squares fitting to obtain the best fit between experimental and predicted strength values.

(2) $$S=S_{\infty }\cdot \exp\left[-{\left(\frac{T}{M}\right)}^{a}\right]$$

Here, S represents the predicted compressive strength (MPa), $S_{\infty }$ is the theoretical ultimate compressive strength of the concrete (MPa), M denotes the maturity index (hours), T is the characteristic time parameter (hours), and a refers to the curve shape parameter. The least-squares method was used to determine the model parameters, yielding the values that provided the best fit to the experimental data.

#### Assumption and Justification of Activation Energy

In maturity-based strength prediction, the apparent activation energy (E_a_) governs the temperature sensitivity of cement hydration and the conversion of temperature history into equivalent age. ASTM C1074 provides an Arrhenius-based framework that allows a single apparent activation energy to be adopted. However, previous studies have shown that E_a_ can vary depending on binder composition and curing temperature, particularly in fly ash-blended concretes under cold-weather conditions [4,13].

In this study, the activation energy was treated as a constant parameter rather than a variable. The objective was not to recalibrate E_a_, but to evaluate the effect of realistic summer, winter-heated, and winter-unheated curing conditions on maturity accumulation and strength development for a consistent mixture. Accordingly, the use of a single activation energy enabled isolation of the temperature history effect and ensured a consistent comparison between curing regimes.

## 3. Results

### 3.1. Early-Age Maturity Development

**Figure 5:**
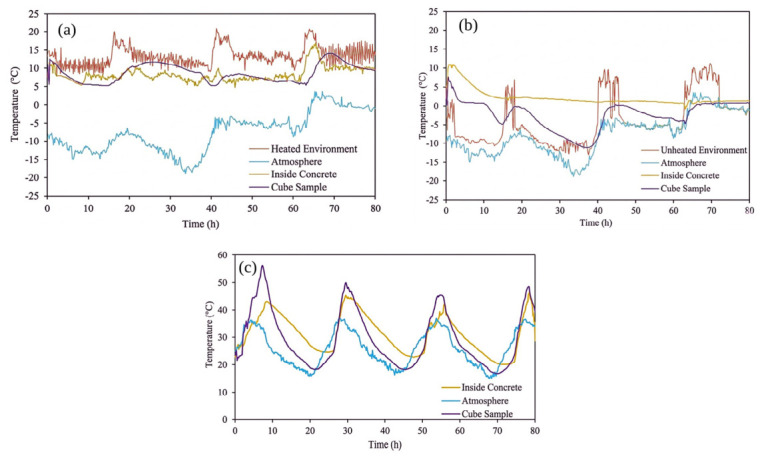
Temperature evolution of SCC under different curing conditions during the first 72 h: (**a**) winter-heated, (**b**) winter-unheated, and (**c**) summer.

The temperature monitoring results indicate that the curing conditions significantly influenced early-age thermal behavior and subsequent maturity development. For example, as shown in Figure 5c, the summer specimens exhibited recurring temperature peaks in the 30–45 °C range due to diurnal fluctuations, which accelerated hydration and led to rapid accumulation of maturity. In comparison, the winter-heated condition (Figure 5a) maintained temperatures slightly above 5 °C within a narrow range. Meanwhile, in the winter-unheated condition (Figure 5b), ambient temperatures reached as low as −18 °C, while internal concrete temperatures remained close to 0 °C. Consequently, these differences in thermal exposure led to markedly different hydration rates.

**Figure 6:**
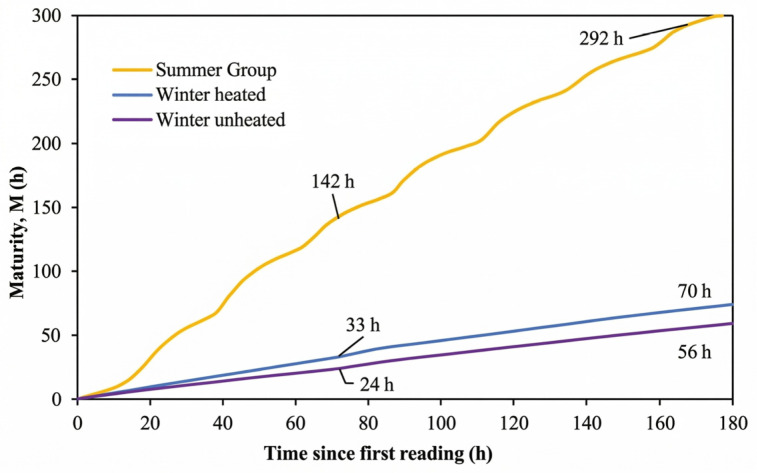
Development of maturity (FHP equivalent age) of SCC under different curing conditions up to 3 and 7 days.

The impact of these thermal regimes is clearly reflected in the maturity development curves (Figure 6). At 72 h, the maturity reached approximately 142 h in the summer specimens, whereas it was limited to 33 h and 24 h in the winter-heated and winter-unheated conditions, respectively. At 7 days, these values increased to 292 h, 70 h, and 56 h, demonstrating substantial differences in maturity accumulation across curing conditions.

A key observation is that the first 72 h accounted for most maturity differences between curing regimes. At this stage, the maturity of summer specimens was about four times higher than that of winter-unheated specimens. This shows that early-age thermal conditions dominate hydration kinetics and control later maturity evolution.

After the first 72 h, both winter-cured samples were exposed to similar conditions. The slopes of the maturity curves then became comparable (Figure 6). Differences observed at later ages were mainly due to the thermal history established during the early-age period, rather than to ongoing differences in curing conditions.

Specimen geometry also influenced thermal behavior, as shown in Figure 5. Cube specimens had greater temperature changes and sometimes dropped below 0 °C. Their higher surface-to-volume ratio made them less stable. Slab specimens kept steadier internal temperatures due to higher thermal mass. Structural dimensions clearly affect early-age thermal response and maturity development.

Overall, results show that maturity development under realistic field conditions is very sensitive to early-age temperature history. Providing enough thermal protection during the first 72 h is critical for hydration and strength development in cold-weather concreting.

### 3.2. Compressive Strength Development

**Figure 7:**
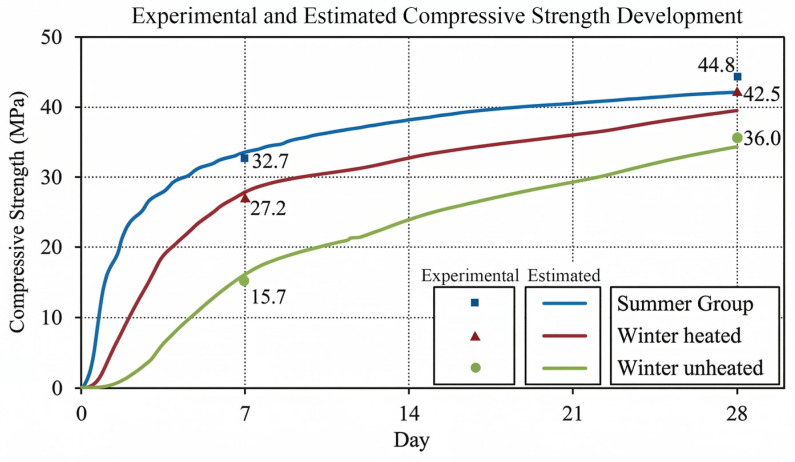
Experimental and maturity-predicted compressive strength development of SCC under different curing conditions.

The compressive strength development of SCC under different curing conditions is shown in Figure 7, along with predictions from the Hansen–Pedersen maturity model. The results indicate that curing temperature had a significant influence on both early-age strength development and long-term performance.

Under summer conditions, the highest strength values recorded were 32.7 MPa at 7 days and 44.8 MPa at 28 days. Maturity-based predictions closely matched experimental results: 33.6 MPa and 42.5 MPa, respectively (R^2^ = 0.92). Strength values are summarized in Table 5. Rapid summer strength gain corresponds with high maturity accumulation reported in Section 3.1. In contrast, under winter-heated conditions, early-age strength was moderately reduced relative to summer: 27.2 MPa at 7 days and 42.5 MPa at 28 days. Predicted strengths (27.9 MPa and 39.9 MPa) also agreed with experimental values (R^2^ = 0.94), as shown in Table 5. This comparison shows that, while controlled winter conditions were cooler than summer conditions, they adequately supported hydration and later-age strength, though early-age strength was reduced.

In contrast, the winter-unheated condition showed markedly slower strength gain, with compressive strengths of 15.7 MPa at 7 days and 36.0 MPa at 28 days (Table 5). Despite this limitation, the maturity model generated precise predictions (16.2 MPa and 34.6 MPa), with the strongest correlation observed in this group (R^2^ = 0.99). This confirms that, even in near-freezing conditions, the maturity method reliably captures the relationship between temperature history and strength development.

The reliability of the maturity-based strength predictions is further supported by the error analysis presented in Table 6. For each curing condition and testing age (7 and 28 days), compressive strength tests were conducted on three cube specimens, and the average values were reported. To assess the predictive performance of the maturity-based model, statistical error metrics including Mean Absolute Error (MAE), Root Mean Square Error (RMSE), Mean Bias Error (MBE), and the coefficient of determination (R^2^) were calculated. MAE and RMSE were used to quantify prediction error, while MBE was used to evaluate the model’s systematic tendency to over- or underestimate compressive strength.

A key observation is that the differences in strength growth between curing conditions match the changes in maturity noted in Section 3.1. The much lower early maturity in the winter-unheated setting led to slower hydration and less strength gain, whereas the greater maturity in the summer and winter-heated settings improved strength.

Overall, the results show that the Hansen–Pedersen maturity model is a strong tool for predicting SCC compressive strength under changing field temperatures. The close match between actual and predicted values, even under tough conditions, supports using maturity to assess strength on site during cold-weather concreting.

### 3.3. Microstructure (SEM) and Phase Analysis (XRD) Comparison

Microstructural observations from scanning electron microscopy analyses revealed that the curing temperature directly influenced both the development of hydration (the chemical reaction that hardens the cement) and the densification of the binder matrix (the compactness and solidity of the material formed). These changes were consistent with the compressive strength results presented in Section 3.2.

**Figure 8:**
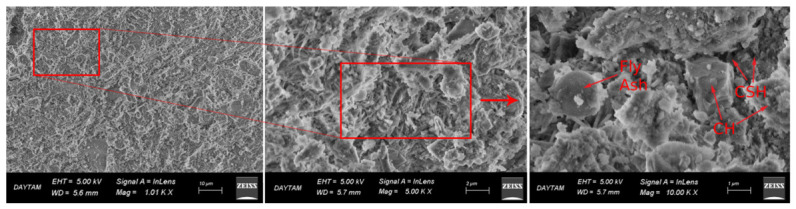
SEM images of SCC specimens cured under summer conditions at 28 days, showing a dense and homogeneous matrix with well-developed C-S-H gel with minimal void content.

These microstructural findings were evident in the summer specimens (Figure 8), where a dense, homogeneous matrix characterized by a well-developed C-S-H network and minimal void content was observed. The efficient progression of hydration reactions under elevated, fluctuating temperatures led to such compactness. This microstructural refinement aligns with previous reports for cementitious systems incorporating supplementary materials under favorable curing conditions [15,16,18]. Consequently, the observed high maturity accumulation and superior compressive strength in this group are directly linked to this advanced hydration state.

**Figure 9:**
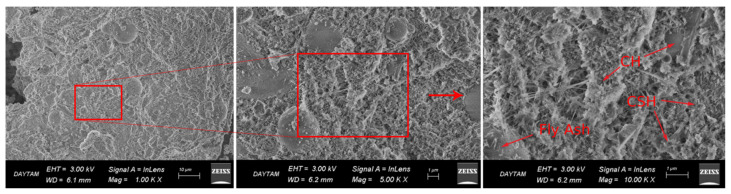
SEM images of SCC specimens cured under winter-heated conditions at 28 days, showing a relatively dense matrix with developed C-S-H gel with limited void content.

In the winter-heated condition (Figure 9), the microstructure exhibited a relatively well-developed C-S-H gel structure with limited voids, although slightly less compact than that of the summer specimens. This suggests that maintaining temperatures above the critical threshold enabled continued hydration and partial microstructural densification, which is consistent with the improved mechanical performance observed at later ages.

**Figure 10:**
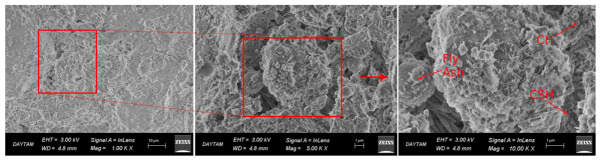
SEM images of SCC specimens cured under winter-unheated conditions at 28 days, showing a porous and heterogeneous matrix with limited C-S-H formation and increased void content.

In contrast, the winter-unheated specimens (Figure 10) had a notably porous, less cohesive microstructure, with more voids and fewer hydration products. The lower presence of C-S-H gel shows that hydration was largely suppressed at low temperatures, hindering full microstructural development. This agrees with studies reporting delayed hydration and reduced phase formation in low-temperature cementitious systems [15,16].

**Figure 11:**
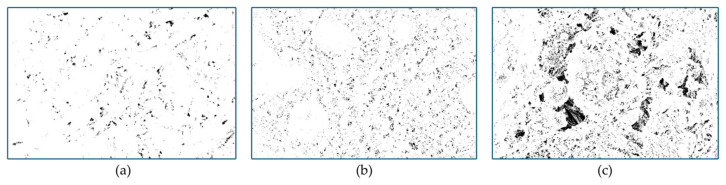
Binary SEM images used for pore area fraction analysis under different curing conditions: (**a**) summer, (**b**) winter-heated, and (**c**) winter-unheated. Black regions represent pores identified through image processing using a constant grayscale threshold (0–70).

To complement the qualitative SEM observations, a quantitative image analysis was performed using ImageJ software. SEM images acquired at 5000× magnification were processed using a constant grayscale threshold (0–70) to identify pore regions. The pore area fraction was calculated as the ratio of the number of pore pixels to the total image area (Figure 11).

The results indicated pore area fractions of approximately 3.53% for the summer specimens, 1.96% for the winter-heated specimens, and 14.90% for the winter-unheated specimens. These values represent image-based pore area fractions rather than absolute volumetric porosity.

The significantly higher pore content observed in the winter-unheated specimens confirms a more porous, less compact microstructure, consistent with the qualitative SEM observations.

**Figure 12:**
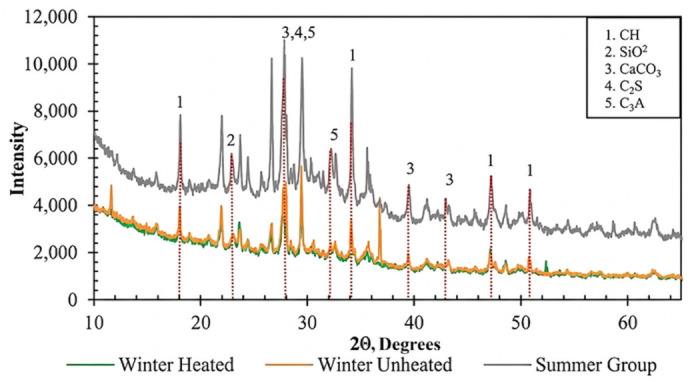
X-ray diffraction (XRD) patterns of SCC specimens cured under summer, winter-heated, and winter-unheated conditions at 28 days, showing variations in portlandite (CH) peak intensities and the broad amorphous hump associated with C–S–H gel formation.

The XRD patterns in Figure 12 support these observations. The summer specimens exhibited higher intensities of portlandite (CH) peaks, particularly around 18° and 34° 2θ. They also showed a more pronounced amorphous hump associated with C-S-H at approximately 29–34° 2θ. These features indicate a more advanced hydration process and a higher degree of phase development. Similar interpretations of CH peak intensity and amorphous phase evolution have been reported in microstructural studies of cement-based materials [19,30].

Winter-heated specimens showed less intense peaks, indicating partial hydration, while winter-unheated specimens had even lower peak intensities and a less distinct amorphous hump, confirming limited phase formation. These XRD findings align with SEM observations and underscore the role of curing temperature in hydration kinetics.

Overall, the combined SEM and XRD analyses show that curing temperature controls hydration kinetics. In turn, hydration kinetics influence phase formation, microstructural densification, and mechanical performance. The strong consistency among microstructural features, maturity development, and compressive strength results confirms that early-age thermal conditions are the dominant factor. These conditions influence both hydration progress and strength development in SCC under realistic field curing conditions.

## 4. Discussion

The results show that early temperature conditions control how quickly fly ash-modified SCC hardens, matures, and gains strength in real field situations. Big differences between summer, winter-heated, and winter-unheated setups show how much cement’s performance depends on temperature, especially during the first 72 h [3,4,5].

The observed performance differences can be evaluated in terms of hydration kinetics, the delayed reactivity of fly ash, and the validity of the maturity approach. The findings show that early-age temperature conditions had a major impact on the hardening, maturity development, and compressive strength of fly ash-modified SCC under field conditions. Summer, winter-heated, and winter-unheated curing regimes differed clearly, particularly during the first 72 h. The markedly reduced strength and maturity observed in the winter-unheated specimens indicate a substantial delay in hydration. This behavior is directly related to the temperature-dependent nature of cement hydration, in which lower temperatures slow reaction kinetics and restrict early C-S-H formation.

This effect becomes more pronounced in fly ash-containing mixtures because of the delayed pozzolanic reactivity of fly ash. At early ages, fly ash contributes little to strength development and mainly acts as a filler, while its beneficial effect emerges later through its reaction with calcium hydroxide [30,31]. Accordingly, the suppression of both cement hydration and fly ash reactivity under low-temperature exposure explains the reduced early-age strength recorded in the winter-unheated group. Previous studies have also shown that while fly ash may reduce early strength, it can improve later-age performance through gradual pozzolanic reactions and enhanced microstructural densification [32].

In addition to delayed hydration, exposure to temperatures near freezing may induce early-age microstructural damage. The freezing of pore water can generate internal stresses, microcracking, interfacial debonding, and increased porosity, which may adversely affect both early- and later-age properties [33,34]. Therefore, the weak performance observed in the winter-unheated specimens should be interpreted not only as a consequence of delayed hydration, but also as a possible result of freeze-related internal damage developing during the early curing period.

In contrast, the improved strength development observed under winter-heated conditions indicates that maintaining suitable curing temperatures can partially compensate for the delayed kinetics of blended systems. Moreover, the high degree of agreement between maturity-predicted and experimentally measured compressive strengths confirms that the Arrhenius-based maturity approach can successfully capture strength development under non-isothermal field conditions. This suggests that maturity-based methods can provide reliable in situ strength estimates when the temperature history is accurately monitored. Nevertheless, slight deviations observed in fly ash-modified mixtures imply that activation energy selection should be interpreted carefully, given the delayed and temperature-sensitive nature of pozzolanic reactions [4,30].

These conclusions are further supported by the microstructural investigations. SEM and XRD analyses showed that higher curing temperatures promoted a denser and more homogeneous microstructure, whereas lower temperatures resulted in a more porous and heterogeneous matrix with limited C-S-H formation. This microstructural evolution directly explains the observed differences in mechanical performance. The denser internal structure developed under favorable curing conditions led to higher strength, whereas increased porosity under cold conditions was associated with weaker behavior. These findings are consistent with previous studies showing that proper curing promotes progressive C-S-H development and matrix refinement, thereby improving mechanical performance [31].

From an engineering perspective, the combined strength and microstructural results suggest that standard laboratory-cured cube specimens may not adequately reflect the actual in situ behavior of concrete under cold-weather field conditions. The thermal gradients and environmental fluctuations encountered in practice differ substantially from laboratory conditions and can significantly influence hydration and strength development. Therefore, reliance solely on laboratory specimens may lead to misleading performance assessments. These findings emphasize the need for thermal control measures, such as heated enclosures, to sustain hydration and ensure sufficient early-age strength during winter concreting.

Overall, the combined evaluation of temperature history, maturity development, compressive strength, and microstructural evolution provides a more realistic understanding of the field performance of fly ash-modified SCC. The results also support the practical use of maturity-based methods as a decision-making tool for cold-weather concreting under non-isothermal curing conditions.

## 5. Conclusions

This study examined how fly ash-modified self-compacting concrete gains strength under seasonal curing. It addresses the limited understanding of how environmental factors affect SCC performance. The approach combined maturity-based analysis and microstructural characterization to bridge this gap.

The main findings can be summarized as follows:

- The temperature during the first 72 h governs concrete strength development under field conditions.Temperature history differed among summer, winter-heated, and winter-unheated settings. These differences led to marked variations in hydration progress and compressive strength.
- Standard cube samples do not reflect real-life cold-weather strength. They lose heat faster and are smaller.
- The maturity method accurately predicts strength under non-isothermal field conditions and reliably estimates in situ strength.
- Microstructural analyses showed that curing temperature directly affects hydration, phase development, and matrix densification. These impact mechanical performance.

Overall, the results confirm that temperature is the dominant parameter controlling early-age behavior in fly ash-modified SCC under field conditions. Inadequate thermal curing significantly delays hydration, maturity development, and early-age strength, whereas controlled heating can effectively mitigate these effects. The combined strength, maturity, and microstructural findings demonstrate that maturity-based approaches provide a practical and reliable basis for estimating in situ strength and supporting decision-making in cold-weather concreting. Future studies should further examine long-term durability under realistic field exposure conditions.

## Figures and Tables

**Table 1:** Physical and chemical properties of the cement.

Cement Component (%)	CEM II/A-LL 42.5 R
SiO_2_	17.89
Al_2_O_3_	4.84
Fe_2_O_3_	3.06
CaO	61.81
MgO	2.89
SO_3_	3.2
Na_2_O	0.31
K_2_O	0.7
CI^−^	0.0058
Residue on 32 μm Sieve (%)	10.3
Specific Gravity (g/cm^3^)	3.06
Specific Surface Area (cm^2^/g)	3668
Initial Setting Time (h–min)	170
Final Setting Time (h–min)	230
Compressive Strength (MPa)	
2nd Day	30
7th Day	43.3
28th Day	54.6

**Table 2:** Properties of the fly ash used in the experimental study.

Parameter	Analysis Method	Unit	Analysis Result
Loss on Ignition (L.O.I)	TS EN 196-2	%	2.40
Chloride Content (by XRF)	TS EN 196-2	%	0.06
Free Lime (Free CaO)	TS EN 451-1	%	0.23
Sulfate (SO_3_)	XRF Method	%	0.98
Total Alkalis	TS EN 196-2	%	2.21
SiO_2_ + Al_2_O_3_ + Fe_2_O_3_	Calculation	%	89.07
Reactive Calcium Oxide (R.CaO)	TS EN 196-2	%	-
Reactive Silicon Oxide (R.SiO_2_)	TS EN 197-1	%	23.54
28-Day Activity Index	TS EN 450-1	%	77.23
90-Day Activity Index	TS EN 450-1	%	93.29
Density Determination	ASTM C188	g/cm^3^	2.34
Fineness Determination (45 µm Sieve Residue)	In-House Method	%	22.30

**Table 3:** Concrete mix design.

w/b Ratio	Water (kg/m^3^)	Cement (kg/m^3^)	Fly Ash (kg/m^3^)	Fine Aggregate (kg/m^3^)	Coarse Aggregate (kg/m^3^)	Superplasticizer (% by Binder)
0.45	225	375	125	805	676	5

**Table 4:** Limits for SCC tests (EFNARC 2005) and test results.

Test	Unit	Min. Value	Max. Value	Test Result
Slump Flow	mm	650	800	760
T50Slump Flow Time	sn	2	5	2.1
V-Funnel	sn	6	12	7.6
L-Box	H2/H1	0.8	1.0	0.96

**Table 5:** Experimental compressive strength results and Hansen–Pedersen model parameters.

	Experimental Compressive Strength Results		Hansen–Pedersen			Estimated Compressive Strength Development		R^2^
	7 Day	28 Day	S∞	Τ	α	7 Day	28 Day	
Summer	32.7	44.8	55	75	0.52	33.6	42.5	0.92
Winter-Heated	27.2	42.5	55	44	0.82	27.9	39.9	0.94
Winter-Unheated	15.7	36.0	55	70	0.90	16.2	34.6	0.99

**Table 6:** Error-based statistical performance of maturity-based strength predictions under different curing conditions.

Curing Condition	MAE (MPa)	RMSE (MPa)	Mean Bias Error, MBE (MPa)
Summer	1.60	1.77	+0.70
Winter-Heated	1.65	1.88	−0.95
Winter-Unheated	0.95	1.04	−0.45

## Data Availability

The original contributions presented in the study are included in the article, further inquiries can be directed to the corresponding author.
